# Early complications associated with fixation constructs of operatively treated patella fractures: a retrospective study

**DOI:** 10.1007/s00590-026-04689-y

**Published:** 2026-02-26

**Authors:** Robin Litten, Anthony Wilson, Doriann Alcaide, Ryan McIlwain, Swapnil Singh, Jonathan Ellis, Joey Johnson, Clay Spitler

**Affiliations:** 1 Department of Orthopaedic Surgery, University of Alabama at Birmingham, Birmingham, USA; 2 Department of Orthopaedic Surgery, University of Missouri–Kansas City, Kansas City, USA; 3 Department of Orthopaedic Surgery, St. Barnabas Hospital, The Bronx, USA; 4 Department of Orthopaedic Surgery, University of Cincinnati Medical Center, Cincinnati, USA

**Keywords:** Patella fracture, Fixation techniques, Symptomatic implant removal, Tension band wiring

## Abstract

**Purpose:**

Although multiple fixation constructs exist for patella fractures, postoperative complications are common, and optimal construct choice remains unclear. This study evaluated construct-specific complication rates in operatively treated patella fractures.

**Methods:**

A retrospective review was conducted of adult patients who underwent surgical fixation of patella fractures (AO/OTA 34) at a Level I trauma center between 2012 and 2022. Patients younger than 18 years, treated with isolated cerclage wiring, or with less than 90 days of follow-up were excluded. Patients were stratified by fixation construct: tension band wiring (TBW), plate, screws, tendon advancement, or multiple constructs. Demographic, injury, and clinical data were obtained from medical records. Primary outcomes included reoperation, fixation failure, nonunion, deep infection, and SIR.

**Results:**

A total of 242 patients were included and stratified by construct type: TBW (*n* = 106), plate (*n* = 18), screws (*n* = 39), TA (*n* = 60), and multiple constructs (*n* = 19). Patients treated with plates were significantly younger (37.1 vs. 45.8 years; *p* = 0.023) and more likely to have open fractures (50.0%; *p* = 0.025), comminuted fracture patterns (≥ 4 fragments: 83.3%; *p* < 0.001), and OTA 34C3 fractures (77.8%; *p* < 0.001). Plate fixation was associated with a higher rate of SIR compared to other groups (27.8%; *p* = 0.012), while no significant differences were observed in reoperation (*p* = 0.142), nonunion (*p* = 0.652), fixation failure (*p* = 0.316), or infection (*p* = 0.505). In subgroup analysis, suture cerclage augmentation in TBW was associated with significantly higher rates of reoperation (85.7% vs. 19.2%; *p* < 0.001), nonunion (57.1% vs. 8.1%; *p* = 0.003), and fixation failure (57.1% vs. 15.2%; *p* = 0.019).

**Conclusion:**

Plate fixation was associated with higher SIR, and suture cerclage in TBW constructs was associated with increased complications in patella fracture fixation.

**Supplementary Information:**

The online version contains supplementary material available at 10.1007/s00590-026-04689-y.

## Introduction

Patella fractures are relatively uncommon injuries, accounting for 1% of all skeletal injuries and usually resulting from direct force to the knee or violent eccentric quadriceps contraction [1]. While immobilization of minimally displaced fractures with intact extensor mechanisms classically yields good outcomes, displaced fractures, open fractures, or fractures with disrupted extensor mechanisms are typically treated with open reduction internal fixation (ORIF) [1–3].

There are several techniques for ORIF of patella fractures, including plates, screws, tension band wiring (TBW), tendon advancements, or patellectomy [4]. TBW constructs, using K-wires or cannulated screws with stainless steel wire or nonabsorbable suture tension band constructs, are widely used to treat patella fractures [1, 4]. These constructs transfer quadriceps tensile forces into compression across the fracture site [1, 4]. Locking plates have been shown to be a viable option for patella fixation, offering reduced interfragmentary gap movement and increased resistance to failure compared to TBW [5]. Fractures associated with poor bone quality and extensive comminution may not be amenable to ORIF, necessitating partial or total patellectomy with tendon advancement [4, 6].

Despite union rates exceeding 95% and infection rates remaining below 5% following ORIF of patellar fractures, other complications are relatively common. For example, symptomatic implant removal (SIR) is reported in up to 50% of cases, and loss of reduction occurs in approximately 5–22% of cases [3, 4, 7, 8]. TBW in particular has been associated with high rates of pain and implant removal, often due to wire migration and hardware prominence [9, 10]. In contrast, plate fixation has demonstrated superior biomechanical strength as well as lower rates of complications and SIR compared to TBW [9, 11–13].

Despite the availability of multiple fixation techniques, the optimal choice of for management of patellar fractures remains unclear, and reoperation rates remain high. Conflicting data regarding TBW, hardware selection, and technical variations have made outcome comparisons challenging. The purpose of this study was to compare outcomes among different fixation techniques in the surgical treatment of patella fractures. The authors hypothesized that plate fixation would be associated with a higher rate of SIR compared to other techniques.

## Methods

### Study design, setting, and participants

Following institutional review board approval, a retrospective review was conducted to identify patients who underwent surgical fixation of patellar fractures (AO/OTA 34) at a single Level I trauma center from 2012 to 2022. Patients were identified using Current Procedural Terminology code 27,524. Patients were excluded if they were under 18 years of age, had less than 90 days of follow-up, were treated with isolated cerclage wiring, or had insufficient medical record documentation. Patients with less than 90 days of follow-up were also included if they experienced a documented postoperative complication within the first 90 days following surgery.

Patient demographic, injury, and clinical data were obtained through retrospective review of the electronic medical record (EMR). For each patient, the type of fixation construct used (tension band wiring [TBW], plate, screws, tendon advancement [TA], or multiple constructs) was recorded. All TBW constructs were performed using screws. Patients classified as undergoing TA uniformly had partial patellectomy with advancement of the patellar tendon to the remaining bone. There were no cases of total patellectomy with TA during the study period.

Demographic variables included age, sex, body mass index (BMI), history of tobacco use, diabetes mellitus, osteoporosis, and American Society of Anesthesiologists (ASA) classification. Osteoporosis was identified by an EMR-recorded diagnosis, consistent with the World Health Organization diagnostic criteria [14], or the presence of active treatment of osteoporosis. Injury-related variables included mechanism of injury, polytrauma status, ipsilateral lower extremity injury, open fracture status, Orthopaedic Trauma Association (OTA) fracture classification, and the degree of comminution (defined as ≥ 4 fracture fragments). Polytrauma was defined as Injury Severity Score (ISS) of > 16 [15, 16]. Clinical outcomes and recovery metrics recorded were follow-up duration (in days), reoperation, SIR, nonunion, fixation failure, deep infection, time from surgery to initiation of range of motion, and time from surgery to full unrestricted range of motion.

Patients were classified based on fixation construct: tension band wiring (TBW), plates (including constructs with plates and associated free screws), screws alone, tendon advancement, or multiple fixation constructs (defined as two or more of the aforementioned methods). The primary outcomes assessed in this study were rates of reoperation, deep infection, failure of fixation requiring revision, SIR, and nonunion. Fixation failure was defined as hardware loosening or breakage necessitating reoperation after initial definitive fixation [17]. Nonunion was defined as radiographic or intraoperative evidence of hypertrophic, oligotrophic, or atrophic nonunion as well as infected osteolysis or synovial pseudoarthrosis persisting at 6 months after index surgery [18, 19]. Deep infection was defined as infection requiring return to the operating room for treatment [20].

## Statistical analyses

Fractures were grouped according to the definitive fixation construct. Descriptive statistics were generated to characterize the study population. Categorical variables are presented as numbers and percentages and were compared using chi-square or Fisher’s exact test, as appropriate. Continuous variables are reported as means with standard deviations and were compared using one-way ANOVA for comparisons involving three or more groups, or independent t-tests for comparisons between two groups. Multivariable binary logistic regression was performed to identify variables independently associated with fixation failure. The threshold for statistical significance was defined as a two-sided* p*-value of ≤ 0.05. All statistical analyses were performed with IBM SPSS (Version 29.0.2.0).

## Results

Of 497 patients with patellar fractures treated operatively at this institution during the study period, 242 met inclusion criteria and were stratified by fixation type: tension band wiring (TBW, *n* = 106), plate (*n* = 18), screws (*n* = 39), tendon advancement (TA, *n* = 60), and multiple constructs (*n* = 19). Among patients treated with multiple constructs, 63.2% underwent TA with screws (*n* = 12), 15.8% received TBW with screws (*n* = 3), 15.8% were treated with plate and TBW (*n* = 3), and 5.3% underwent plate with TA (*n* = 1) (SDC Table 1). Among patients treated with plate fixation, the most common construct was an anatomic plate (*n* = 14, 77.8%), followed by mini-fragment (*n* = 2, 11.1%) and mesh plates (*n* = 2, 11.1%) (SDC Table 2).

**Table 1:** Demographic and clinical characteristics of patients undergoing operative fixation of patella fractures, stratified by construct type

Variable *n* (%) or mean (SD)	TBW *n* = 106	Plate *n* = 18	Screws *n* = 39	TA *n* = 60	Multiple *n* = 19	*p*-value
Age (years)	49.0 (18.8)	37.1 (16.7)	42.5 (16.5)	43.5 (18.2)	50.6 (14.5)	**0.023**
Follow-up (days)	336.1 (391.7)	255.5 (128.0)	264.1 (166.4)	297.8 (239.8)	295.4 (208.2)	0.667
Male sex	60 (56.6)	11 (61.1)	24 (61.5)	31 (51.7)	12 (63.2)	0.836
BMI (kg/m^2^)	27.6 (7.4)	29.6 (7.0)	27.1 (4.3)	28.2 (6.3)	28.6 (4.2)	0.637
Tobacco use	30 (28.3)	9 (50.0)	14 (35.9)	24 (40.0)	9 (47.4)	0.219
DM	15 (14.2)	1 (5.6)	4 (10.3)	7 (11.7)	2 (10.5)	0.856
Osteoporosis	21 (19.8)	2 (11.1)	5 (12.8)	5 (8.3)	1 (5.3)	0.204
ASA score						0.336
I	4 (3.8)	2 (11.1)	2 (5.1)	1 (1.7)	1 (5.3)	
II	31 (29.2)	10 (55.6)	20 (51.3)	21 (35.0)	6 (31.6)	
III	62 (58.5)	4 (22.2)	14 (35.9)	30 (50.0)	10 (52.6)	
IV	8 (7.5)	2 (11.1)	3 (7.7)	8 (13.3)	2 (10.5)	
V	1 (0.9)	0 (0.0)	0 (0.0)	0 (0.0)	0 (0.0)	

SD,  standard deviation; TBW , tension band wiring; TA ,  tendon advancement; multiple , multiple fixation techniques; BMI , body mass index; kg/m^2^,  kilograms per meter squared; DM , diabetes mellitus; ASA , American Society of Anesthesiologists

Values presented in bold indicate statistical signifi cance at p 0.05

**Table 2:** Injury patterns and fracture characteristics stratified by fixation construct

Variable *n* (%) or mean (SD)	TBW *n* = 106	Plate *n* = 18	Screws *n* = 39	TA *n* = 60	Multiple *n* = 19	*p*-value
Polytrauma	26 (24.5)	8 (44.4)	14 (35.9)	23 (38.3)	6 (31.6)	0.249
Ipsilateral LE injury	43 (40.6)	11 (61.1)	19 (48.7)	30 (50.0)	9 (47.4)	0.490
Open injury	22 (20.8)	9 (50.0)	12 (30.8)	15 (25.0)	9 (47.4)	**0.025**
≥ 4 fragments	55 (51.9)	15 (83.3)	15 (38.5)	26 (43.3)	16 (84.2)	**< 0.001**
OTA classification						**< 0.001**
34C1	24 (22.6)	1 (5.6)	5 (12.8)	8 (13.3)	2 (10.5)	
34C2	18 (17.0)	1 (5.6)	8 (20.5)	4 (6.7)	2 (10.5)	
34C3	63 (59.4)	14 (77.8)	21 (53.8)	30 (50.0)	15 (78.9)	
34 A	0 (0.0)	0 (0.0)	0 (0.0)	15 (25.0)	0 (0.0)	
34B	1 (0.9)	2 (11.1)	5 (12.8)	3 (5.0)	0 (0.0)	
Mechanism of injury						0.179
MVC	56 (52.8)	12 (66.7)	22 (56.4)	42 (70.0)	11 (57.9)	
MCC	6 (5.7)	0 (0.0)	3 (7.7)	0 (0.0)	3 (15.8)	
GSW	1 (0.9)	1 (5.6)	2 (5.1)	2 (3.3)	1 (5.3)	
FFS	37 (34.9)	2 (11.1)	10 (25.6)	13 (21.7)	3 (15.8)	
FFH	2 (1.9)	1 (5.6)	1 (2.6)	2 (3.3)	1 (5.3)	
Other	4 (3.8)	2 (11.1)	1 (2.6)	1 (1.7)	0 (0.0)	

SD , standard deviation; TBW , tension band wiring; TA , tendon advancement; multiple , multiple fixation techniques, LE , lower extremity; OTA , Orthopaedic Trauma Association; MVC , motor vehicle collision; MCC , motorcycle collision; GSW , gunshot wound; FFS , fall from standing; FFH , fall from height

Values presented in bold indicate statistical signifi cance at p 0.05

The average age of this cohort was 45.8 (range: 17–89 years), with 56.8% identifying as male. Patients treated with plates were significantly younger than those in other fixation groups (mean age 37.1; *p* = 0.023). The mean BMI was 27.8 kg/m^2^ (range: 17.0–53.0 kg/m^2^). The mean follow-up of the cohort was 305.8 days (range: 92-2185 days) and did not differ significantly when stratified by construct type (*p* = 0.667). Additionally, there were no significant differences in sex (*p* = 0.836), BMI (*p* = 0.637), tobacco use (*p* = 0.219), diabetes (*p* = 0.856), or osteoporosis (*p* = 0.204) across fixation groups (Table 1).

Fracture patterns and injury characteristics are presented in Table 2. Among the 242 patients in this cohort, 27.7% (*n* = 66) had an open fracture. When stratified by fixation construct, the proportion of patients with open fractures was highest within the plate group (9/18; 50.5%) and the multiple constructs group (9/19; 47.4%) (*p* = 0.025) (Table 2). A total of 127 patients (52.5%) had comminuted fracture patterns with ≥ 4 fragments. When this was stratified by fixation construct, a significantly higher proportion of fractures treated with plates (15/18; 83.3%) or multiple constructs (16/19; 84.2%) involved ≥ 4 fragments compared to other fixation methods (*p* < 0.001) (Table 2). OTA classification also differed significantly across groups (*p* < 0.001), with plates (14/18; 77.8%) and multiple constructs (15/19; 78.9%) used most frequently in type 34C3 fractures (Table 2).

Clinical outcomes and recovery data are shown in Table 3. Among the entire cohort of 242 patients, the overall rate of SIR in this cohort was 8.3%. When stratified by fixation construct, the rate of SIR was significantly higher in plate group compared to all other groups (27.8%; *p* = 0.012) (Table 3). The overall rate of nonunion was 9.5%, deep infection was 8.6%, failure of fixation was 14.8%, and reoperation was 22.2%. No significant differences were observed in reoperation rates (*p* = 0.142), nonunion (*p* = 0.652), fixation failure (*p* = 0.316), deep infection (*p* = 0.505) when stratified by fixation construct (Table 3). The average time to full final knee range of motion was 89.8 days (range: 1-362 days). No significant differences were observed when stratified by fixation construct (*p* = 0.489) (Table 3).

**Table 3:** Clinical outcomes and postoperative recovery by fixation construct

Variable *n* (%) or mean (SD)	TBW *n* = 106	Plate *n* = 18	Screws *n* = 39	TA *n* = 60	Multiple *n* = 19	*p*-value
Reoperation	25 (23.6)	5 (27.8)	6 (15.4)	10 (16.7)	8 (42.1)	0.142
SIR	10 (9.6)	5 (27.8)	3 (7.7)	1 (1.7)	1 (5.3)	**0.012**
Nonunion	12 (11.3)	1 (5.9)	2 (5.1)	5 (8.5)	3 (15.8)	0.652
Fixation failure	19 (17.9)	2 (11.1)	4 (10.3)	6 (10.0)	5 (26.3)	0.316
Deep infection	9 (8.5)	2 (11.1)	1 (2.6)	6 (10.0)	3 (15.8)	0.505
Surgery to full ROM (days)*	93.8 (39.8)	104.3 (54.6)	84.8 (37.3)	100.9 (65.0)	88.6 (26.1)	0.489

SD , standard deviation; TBW , tension band wiring; TA , tendon advancement; multiple , multiple fixation techniques; ROM , range of motion; SIR , symptomatic implant removal

*Number of days from the date of surgery to when the patient was released to perform full, unrestricted range of motion

Values presented in bold indicate statistical signifi cance at p 0.05

In a subgroup analysis of TBW constructs, use of a suture cerclage was associated with significantly higher rates of reoperation (85.7% vs. 19.2%; *p* < 0.001), nonunion (57.1% vs. 8.1%; *p* = 0.003), and fixation failure (57.1% vs. 15.2%; *p* = 0.019) (Table 4). In addition, comminution with ≥ 4 fracture fragments was significantly more common in the suture cerclage group (71.4% vs. 50.5%; *p* = 0.046) (Table 4). Although not statistically significant, higher rates of SIR (28.6% vs. 8.2%; *p* = 0.135) and deep infection (28.6% vs. 7.1%; *p* = 0.108) were also noted in the suture cerclage group (Table 4).

**Table 4:** Comparison of outcomes in TBW constructs with and without suture cerclage

Outcome	No suture (*n* = 99)	Suture cerclage (*n* = 7)	*p*-value
Reoperation	19 (19.2)	6 (85.7)	**< 0.001**
SIR	8 (8.2)	2 (28.6)	0.135
Nonunion	8 (8.1)	4 (57.1)	**0.003**
Failure of fixation	15 (15.2)	4 (57.1)	**0.019**
Deep infection	7 (7.1)	2 (28.6)	0.108
≥ 4 fragments	50 (50.5)	5 (71.4)	0.046

TBW , tension band wiring, SIR , symptomatic implant removal

Values presented in bold indicate statistical signifi cance at p 0.05

When stratified by metallic wire and heavy nonabsorbable braided suture TBW constructs, there were no statistically significant differences in complication rates (Table 5). Rates of reoperation were similar between groups (26.9% vs. 28.8%, *p* = 0.777), as were rates of SIR (11.5% vs. 11.3%, *p* = 0.482), failure of fixation (19.2% vs. 23.8%, *p* = 0.931), and deep infection (7.7% vs. 12.5%, *p* = 0.501) (Table 5). Although nonunion was more frequently observed in the nonabsorbable braided suture TBW group (17.5% vs. 3.8%), this difference did not reach statistical significance (*p* = 0.119) (Table 5).

**Table 5:** Comparison of outcomes between stainless steel wire and heavy nonabsorbable braided suture TBW constructs

Outcome	Stainless steel wire (*n* = 26) *n* (%)	Nonabsorbable braided suture (*n* = 80) *n* (%)	*p*-value
Reoperation	7 (26.9)	23 (28.8)	0.777
SIR	3 (11.5)	9 (11.3)	0.482
Nonunion	1 (3.8)	14 (17.5)	0.119
Failure of fixation	5 (19.2)	19 (23.8)	0.931
Deep infection	2 (7.7)	10 (12.5)	0.501

TBW , tension band wiring; SIR , symptomatic implant removal

On multivariable logistic regression analysis (Table 6), none of the fixation constructs (plate vs. TBW, screws vs. TBW, tendon advancement vs. TBW, multiple fixation techniques vs. TBW), age, presence of an open fracture, or comminution with ≥ 4 fragments were independently associated with fixation failure.

**Table 6:** Multivariable logistic regression evaluating factors associated with fixation failure

Variable	Odds ratio (95% CI)	*p*-value
Age (per year increase)	0.999 (0.979–1.020)	0.946
Plate vs. TBW	0.590 (0.118–2.945)	0.520
Screws vs. TBW	0.576 (0.180–1.843)	0.352
TA vs. TBW	0.531 (0.197–1.427)	0.209
Multiple vs. TBW	1.725 (0.528–5.634)	0.366
Open fracture	0.515 (0.199–1.331)	0.171
≥ 4 fracture fragments	1.498 (0.690–3.254)	0.307

TBW , tension band wiring, TA , tendon advancement, multiple , multiple fixation techniques, CI , confidence interval

## Discussion

In this retrospective cohort of 242 operatively treated patella fractures, SIR occurred significantly more often in the plate fixation group compared to other constructs. Among TBW constructs, suture cerclage augmentation was associated with higher rates of reoperation, nonunion, and fixation failure; however, this technique was more frequently used in the most complex fracture patterns. No statistically significant differences were observed between fixation methods in rates of reoperation, nonunion, fixation failure, deep infection, or time to full range of motion. Overall, these findings suggest that the fixation techniques analyzed in this study produced comparable clinical outcomes.

There were no statistically significant differences between fixation constructs in terms of reoperation, nonunion, failure of fixation, deep infection, time to initiation of knee motion, or time to full range of motion. The nonunion rate observed in this study (9.4%) aligns with rates currently reported in the literature (1.3–12.5%) [7, 21]. The reoperation rate (22.2%) is lower than previously reported rates (33.6%), whereas the infection rate (8.6%) exceeds that reported in the literature (3.2%) [7]. The observed fixation failure rate (14.8%) falls within the range of current literature (0–60%) [4, 7, 22]. However, direct comparisons across studies are limited by differences in cohort size, follow-up duration, as well as different definitions of infection, fixation failure, and nonunion across studies. Taken together, these findings suggest that the various fixation techniques yielded comparable clinical outcomes.

Patients treated with plates or multiple constructs had the highest proportion of open fractures and comminuted fracture patterns. Notably, OTA type 34C3 fractures were most commonly managed with these fixation methods. This is consistent with a systematic review by Raja et al., which identified OTA type 34C3 fractures as the most frequent indication for plate fixation [23]. Similarly, Hargett et al. reported that plate fixation is a viable option for comminuted fractures, describing their preferred technique as a lateral parapatellar approach with interfragmentary screw fixation and a minifragment locked neutralization plate [9]. The proliferation of minifragment fixation systems and anatomic plates has facilitated broader use of this technique.

The highest rate of SIR in this study was observed in the plate fixation group. However, differences in group sizes across fixation methods may influence the observed rates in this study. Additionally, plates were frequently used in patients with more severe fracture patterns, including a higher proportion of open injuries, comminution with ≥ 4 fragments, and OTA 34C3 fractures. In this context, the increased likelihood of later implant removal could represent a trade-off for achieving stable fixation and union in complex fractures that might otherwise require patellectomy or carry a high risk of mechanical failure. Despite being used in the most complex injuries, plate constructs did not demonstrate higher rates of nonunion, fixation failure, reoperation, or deep infection compared with other fixation methods.

The overall rate of SIR in this study (8.3%) is lower than currently reported rates for SIR (10–50%) [22]. Greenberg et al. reported that, following patella ORIF, SIR significantly improves patient-reported pain and quality of life outcomes but not functional outcomes [22]. While their findings underscore the potential benefit of SIR in select patients, patient-reported outcomes following implant removal were not assessed in this current study. This represents an opportunity for future research to evaluate the impact of SIR on long-term functional recovery, pain, and quality of life.

In a sub analysis of patients treated with TBW with or without suture cerclage, patients treated with suture cerclage demonstrated significantly higher rates of reoperation, nonunion, and fixation failure compared to those treated without suture cerclage. Notably, the presence of ≥ 4 fracture fragments was significantly more common in the suture cerclage group, suggesting that fracture complexity may have contributed to both construct selection and the observed outcomes. Prior studies have similarly utilized cerclage-based techniques in the setting of highly comminuted patellar fractures, supporting its role in managing complex injury patterns [24, 25]. These findings highlight that suture cerclage techniques may be employed in the setting of greater fracture complexity, which may confound comparisons of outcomes across fixation constructs.

When comparing TBW constructs using metallic wire versus heavy nonabsorbable braided suture, no significant differences in complication rates were observed. However, multiple studies, including two meta-analyses and a retrospective series, have reported lower rates of reoperation and implant-related complications with suture-based constructs compared to metallic fixation [26–28]. Although this study did not demonstrate statistically significant differences, these findings suggest potential advantages of suture-based TBW that warrant further investigation.

Multivariable logistic regression analysis did not identify any statistically significant predictors of fixation failure among factors such as age, fracture characteristics, or fixation method (plate vs. TBW, screws vs. TBW, tendon advancement vs. TBW, multiple fixation techniques vs. TBW). Because fixation method was not identified as an independent predictor of fixation failure, these findings may suggest that the contemporary fixation techniques evaluated in this study provide broadly comparable mechanical stability, even in the setting of comminuted or open fractures. By capturing a broad spectrum of fixation strategies and fracture patterns, this study adds valuable insight to an area with limited comparative data and lack of consensus. These findings may help guide implant selection and technical decision-making in the operative management of patella fractures.

## Limitations

This study has limitations that should be considered when interpreting these findings, including those inherent to its retrospective design. This study was conducted at a single institution, which may limit generalizability to other settings with different patient populations, clinical protocols, or patellar fracture management practices. A substantial number of operatively treated patella fractures were excluded (255 of 497; 51.3%) due to limited follow-up, isolated cerclage fixation, age criteria, or incomplete documentation, which may introduce selection bias and affect the generalizability of these findings. Additionally, there were discrepancies in group sizes across fixation methods, which could have limited the ability to detect differences between groups. The selection of a fixation method was influenced by both surgeon preference and clinical context, which may have introduced variability in treatment choice. Interpretation of outcomes related to cerclage suture augmentation should be made with caution. Only a small proportion of patients treated with TBW underwent cerclage augmentation (7 of 106), limiting the ability to draw meaningful conclusions about its impact. Moreover, cerclage was more commonly applied in fractures with greater complexity, suggesting that the higher complication rates observed are more likely a reflection of case severity and selection bias rather than a causal relationship between cerclage and poor outcomes. As such, these findings warrant further investigation in larger cohorts before making definitive statements about the role of cerclage in patella fracture fixation.

## Conclusion

In this retrospective study of operatively treated patella fractures, plate fixation was associated with a significantly higher rate of SIR compared to other fixation methods. Suture cerclage augmentation in TBW constructs was associated with higher rates of reoperation, nonunion, and fixation failure, though appeared to be used in the most complex fracture patterns. Among TBW constructs, no significant differences in outcomes were observed between heavy nonabsorbable braided suture and stainless-steel wire, suggesting that either may be appropriate for patella fracture fixation. No independent risk factors for fixation failure were identified. Individualized implant selection is recommended for optimal fracture management.

## Supplementary Information

Below is the link to the electronic supplementary material.

Supplementary file1

## Data Availability

No datasets were generated or analysed during the current study.
