# Supplementary material for: Early complications associated with fixation constructs of operatively treated patella fractures: a retrospective study
Source: Eur J Orthop Surg Traumatol. 2026 Feb 26;36(1):118. doi: 10.1007/s00590-026-04689-y (PMC12946273; doi:10.1007/s00590-026-04689-y)
Supplement: Supplementary file 1 — Supplementary file1 [file 590_2026_4689_MOESM1_ESM.docx]

**SUPPLEMENTAL DIGITAL CONTENT**

SDC Table 1. Techniques used in patients with multiple fixation constructs (n = 19).

| Technique | Count | Percent |
| --- | --- | --- |
| Tendon advancement with screws | 12 | 63.2 |
| Tension band wiring with screws | 3 | 15.8 |
| Tension band wiring with plate | 3 | 15.8 |
| Plate with tendon advancement | 1 | 5.3 |

SDC Table 2. Distribution of plate types utilized in the operative fixation of patella fractures (n = 18).

| Plate type | Count | Percent |
| --- | --- | --- |
| Mini fragment | 2 | 11.1 |
| Anatomic | 14 | 77.8 |
| Mesh | 2 | 11.1 |
| Total | 18 | 100.0 |
